# Supplementary material for: Diurnal variation of islet autoantibody titers in established type 1 diabetes suggests restricted-time sampling improves aab measurement and detection
Source: Sci Rep. 2026 Oct 29;16:35742. doi: 10.1038/s41598-026-72569-w (PMC13719494; doi:10.1038/s41598-026-72569-w)
Supplement: Supplementary file 1 [file 41598_2026_72569_MOESM1_ESM.docx]

**Supplemental Table 1: Acrophase Estimates (95% Confidence Interval) of Immunoglobulins (Ig) in Stage 4 Type 1 Diabetes and Controls**

|  | **Control** | | | | |  |  | **T1D** | | | | |
| --- | --- | --- | --- | --- | --- | --- | --- | --- | --- | --- | --- | --- |
|  |  |  |  | **95% CI** | |  |  |  |  |  | **95% CI** | |
|  | **p** | **Acrophase** | **SE** | **lower** | **upper** |  |  | **p** | **Acrophase** | **SE** | **lower** | **upper** |
| **IgM** | 0.0099 | 11:20 | 1.6 | 8:12 | 14:24 |  | **IgM** | 0.0076 | 12:12 | 1.5 | 9:18 | 15:06 |
| **IgG1** | 0.0825 | 18:18 | 3.6 | 11:12 | 1:24 |  | **IgG1** | 0.0012 | 11:54 | 1.2 | 9:36 | 14:18 |
| **IgG2** | <0.0001 | 14:06 | 0.8 | 12:30 | 15:42 |  | **IgG2** | 0.0027 | 13:06 | 1.3 | 10:36 | 15:36 |
| **IgG3** | 0.2459 | 1:36 | 8.6 | 8:48 | 18:18 |  | **IgG3** | 0.0006 | 14:06 | 0.9 | 12:18 | 15:54 |
| **IgG4** | 0.1323 | 17:36 | 3.4 | 10:54 | 0:18 |  | **IgG4** | <0.0001 | 12:06 | 0.8 | 10:30 | 13:42 |
| **IgA1** | 0.0035 | 12:06 | 1.4 | 9:24 | 14:48 |  | **IgA1** | 0.0004 | 9:48 | 0.8 | 8:00 | 11:12 |
| **IgA2** | 0.5242 | 6:48 | 6.1 | 19:48 | 18:48 |  | **IgA2** | 0.0003 | 11:36 | 1.0 | 9:36 | 13:36 |

**“p” refers to Circadian Model significance. “Acrophase” is the estimated time of peak titer. “SE” =Standard Error based on 10,000 Bootstrapped replicates. “95% CI” =95% confidence interval based on 10,000 Bootstrapped replicates.**

**Supplemental Table 2: Acrophase Estimates (95% Confidence Interval) of Autoantibodies (Aab) Having Significant Circadian Variation in Stage 4 Type 1 Diabetes**

|  |  |  |  | **95% CI** | |
| --- | --- | --- | --- | --- | --- |
| **Aab** | **p†** | **Acrophase** | **SE** | **lower** | **upper** |
| **IA** | 0.0028 | 4:08 | 1.3 | 1:35 | 6:41 |
| **ZnT8RA** | 0.0057 | 7:00 | 1.5 | 4:05 | 9.56 |
| **ZnT8WA** | <0.0001 | 8:54 | 1.7 | 5:34 | 12:14 |

**“p” refers to Circadian Model significance. “Acrophase” is the estimated time of peak titer. “SE” =Standard Error based on 10,000 Bootstrapped replicates. “95% CI” =95% confidence interval based on 10,000 Bootstrapped replicates.**

**Supplemental Table 3 Median Aab Standard Deviation (SD) Ratio: Restricted-Time to Random-Time Sample Selection**

|  | **Time of Sampling** | | |
| --- | --- | --- | --- |
| **Aab** | **09:00** | **13:00** | **17:00** |
| **GADA** | 0.998^#^ (0.771,1.477) | ***0.768†***  ***(0.275,0.999)*** | 0.982  (0.072,1.150) |
| **IA** | 1.065  (0.954,1.209) | ***0.905†***  ***(0.760,0.974)*** | ***0.892†***  ***(0.591,0.993)*** |
| **IA2A** | 1.000  (0.434,1.327) | 1.084  (0.895,1.529) | 0.835  (0.578,1.072) |
| **ZNT8RA** | 1.000  (0.622,1.203) | ***0.663†***  ***(0.200,0.839)*** | 0.899  (0.471,1.087) |
| **ZNT8WA** | 1.089  (0.434,1.462) | 1.019  (0.562.1.430) | 1.93  (0.800.5.249) |

^#^Median SD ratio (95% Bootstrap Confidence Interval) of 10,000 Bootstrap Simulations.

†Statistically significantly less than Random Selection SD at p<0.05 based on confidence interval.

**Supplemental Table 4: Comparison of Duration Between Significant Age-TOD Subgroups**

| **Aab (Ln)** | **Age (yrs.)** | ***TOD†***  **Mean Duration (yrs.)** | | **p-value** |
| --- | --- | --- | --- | --- |
| **IA** |  |  |  |  |
|  |  | ***<=14:00*** | ***>14:00*** |  |
|  | **14-19** | 4.19 | 5.18 | 0.6036 |
| **IA-2A** |  | ***<=8:00*** | ***>8:00*** |  |
|  | ***<= 16*** | 0.98 | 3.23 | 0.0818 |
|  | ***17-42*** | 7.10 | 7.52 | 0.6233 |
|  |  |  |  |  |
| **ZnT8A** |  | ***<=9:00*** | ***>9:00*** |  |
|  | ***<=15*** | 1.36 | 3.28 | 0.1463 |
|  |  | ***<=14:00*** | ***>14:00*** |  |
|  | ***26-57*** | 12.58 | 13.32 | 0.7370 |

**†*TOD=Time of Day. p-value from ANOVA.***

**Supplementary Figure 1 CART for GADA and IA**

**GADA**

**IA**

**Age and Time-of-Day (TOD) Subgroups Identified by Machine Learning**

Results are presented for GADA and IA. Top part of diagram of each figure presents the decision tree created by machine learning (CART). Lower part of diagram present boxplots of Ln GADA or Ln IA titers. Median titer is represented by horizontal bar. Analysis of GADA found no subgroups with TOD differences. Analysis of Ln IA found that for subjects 14-19 years old sampling before 2 pm lead to significantly higher titer levels than if sampled after 2 pm (p=0.0097 by ANOVA).

**Supplemental Figure 2 Distribution of Islet Antibody Titer by Sampling Times**

**Supplemental Figure 3 Diurnal Variation Often Exceeds Inter-Assay Variation**

A “Diurnal Coefficient of Variation” (“dCV”) was calculated within each subject using the standard deviation and mean of six titer measurements separated by 4 hours. The ratio of dCV to the inter-assay CV assessed by the TrialNet laboratory (“TNCV”) is plotted (“inter-assay CV ratio”) by antibody (i.e., CV ratio=dCV/TNCV). Short horizontal lines represent median CV ratio. A horizontal reference line (dashed) at the value “1” indicates that dCV=TNCV.

**Supplemental Methods**

**RBA**

The RBA showed good performance in the 2015, 2018, 2020 and 2023 Islet Autoantibody Standardization Program (IASP) workshops, following local or harmonized methods(1; 2). IASP2023 assay sensitivity at 95% specificity was >=62% (IA: 72%, GADA: 88%, IA-2A: 80%, ZnT8RA: 66% and ZnT8WA: 62%). Harmonized thresholds are used for comparison between laboratories including multi-center studies such as The Environmental Determinants of Diabetes in the Young (TEDDY) study(3), while local thresholds are based on assay performance within the single center. Local and harmonized thresholds in serum: IA (0.2 vs 0.95 respectively), GADA (13.5 vs 33 respectively) and IA-2A (1.4 vs 5 respectively). Only local thresholds for ZnT8RA and ZnT8WA (both 1.8) are used as no harmonization studies have been performed. Medium local QC CV% (IA: 17.4, GADA: 14.02, IA-2A: 24.37, ZnT8RA: 19.26; ZnT8WA: 17.94).

**References:**

1. Bonifacio E, Yu L, Williams AK, Eisenbarth GS, Bingley PJ, Marcovina SM, Adler K, Ziegler AG, Mueller PW, Schatz DA, Krischer JP, Steffes MW, Akolkar B. Harmonization of glutamic acid decarboxylase and islet antigen-2 autoantibody assays for national institute of diabetes and digestive and kidney diseases consortia. J Clin Endocrinol Metab 2010;95:3360-3367

2. Long AE, Gooneratne AT, Rokni S, Williams AJ, Bingley PJ. The role of autoantibodies to zinc transporter 8 in prediction of type 1 diabetes in relatives: lessons from the European Nicotinamide Diabetes Intervention Trial (ENDIT) cohort. J Clin Endocrinol Metab 2012;97:632-637

3. Krischer JP, Lynch KF, Schatz DA, Ilonen J, Lernmark Å, Hagopian WA, Rewers MJ, She JX, Simell OG, Toppari J, Ziegler AG, Akolkar B, Bonifacio E, Group TS. The 6 year incidence of diabetes-associated autoantibodies in genetically at-risk children: the TEDDY study. Diabetologia 2015;58:980-987

**Supplemental Attachment: Output from CART**

```
[1] "gad"

Model formula:
gad_ln ~ draw_time + Age_at_Draw

Fitted party:
[1] root
|   [2] Age_at_Draw >= 52.5
|   |   [3] draw_time < 10.5: 2.805 (n = 10, err = 45.1)
|   |   [4] draw_time >= 10.5
|   |   |   [5] Age_at_Draw < 56.5: 3.184 (n = 9, err = 107.7)
|   |   |   [6] Age_at_Draw >= 56.5: 4.275 (n = 11, err = 41.1)
|   [7] Age_at_Draw < 52.5
|   |   [8] Age_at_Draw < 18.5
|   |   |   [9] Age_at_Draw >= 14.5: 4.005 (n = 106, err = 438.1)
|   |   |   [10] Age_at_Draw < 14.5: 4.429 (n = 204, err = 631.6)
|   |   [11] Age_at_Draw >= 18.5
|   |   |   [12] draw_time >= 11.5: 4.276 (n = 89, err = 317.4)
|   |   |   [13] draw_time < 11.5: 4.643 (n = 220, err = 851.5)

Number of inner nodes:    6
Number of terminal nodes: 7
[1] "miaa"

Model formula:
miaa_ln ~ draw_time + Age_at_Draw

Fitted party:
[1] root
|   [2] Age_at_Draw >= 19.5
|   |   [3] Age_at_Draw < 43.5
|   |   |   [4] Age_at_Draw >= 23.5: -2.616 (n = 220, err = 820.1)
|   |   |   [5] Age_at_Draw < 23.5: -1.974 (n = 40, err = 82.5)
|   |   [6] Age_at_Draw >= 43.5
|   |   |   [7] draw_time < 9.5: -2.475 (n = 23, err = 90.7)
|   |   |   [8] draw_time >= 9.5: -1.680 (n = 63, err = 252.8)
|   [9] Age_at_Draw < 19.5
|   |   [10] Age_at_Draw >= 13.5
|   |   |   [11] draw_time >= 14.5: -2.354 (n = 24, err = 89.7)
|   |   |   [12] draw_time < 14.5: -1.388 (n = 134, err = 336.9)
|   |   [13] Age_at_Draw < 13.5
|   |   |   [14] draw_time >= 15.5: -1.489 (n = 8, err = 40.8)
|   |   |   [15] draw_time < 15.5: -0.827 (n = 189, err = 419.2)

Number of inner nodes:    7
Number of terminal nodes: 8
[1] "ia2a"

Model formula:
ia2_ln ~ draw_time + Age_at_Draw

Fitted party:
[1] root
|   [2] Age_at_Draw >= 42.5
|   |   [3] Age_at_Draw >= 55.5: 0.009 (n = 15, err = 98.7)
|   |   [4] Age_at_Draw < 55.5
|   |   |   [5] Age_at_Draw < 43.5: 0.608 (n = 7, err = 10.6)
|   |   |   [6] Age_at_Draw >= 43.5: 2.810 (n = 32, err = 233.3)
|   [7] Age_at_Draw < 42.5
|   |   [8] Age_at_Draw >= 16.5
|   |   |   [9] draw_time >= 8.5: 3.151 (n = 171, err = 1212.4)
|   |   |   [10] draw_time < 8.5: 3.855 (n = 65, err = 341.9)
|   |   [11] Age_at_Draw < 16.5
|   |   |   [12] draw_time >= 8.5: 4.030 (n = 199, err = 877.9)
|   |   |   [13] draw_time < 8.5: 5.284 (n = 31, err = 27.2)

Number of inner nodes:    6
Number of terminal nodes: 7
[1] "znt8"

Model formula:
ZnT8_ln ~ draw_time + Age_at_Draw

Fitted party:
[1] root
|   [2] Age_at_Draw >= 25.5
|   |   [3] Age_at_Draw >= 58: -5.773 (n = 9, err = 9.3)
|   |   [4] Age_at_Draw < 58
|   |   |   [5] draw_time < 14.5: -4.242 (n = 184, err = 688.2)
|   |   |   [6] draw_time >= 14.5: -3.074 (n = 13, err = 71.1)
|   [7] Age_at_Draw < 25.5
|   |   [8] Age_at_Draw >= 15.5
|   |   |   [9] draw_time < 10.5: -3.566 (n = 62, err = 266.9)
|   |   |   [10] draw_time >= 10.5: -2.949 (n = 69, err = 259.5)
|   |   [11] Age_at_Draw < 15.5
|   |   |   [12] draw_time >= 9.5: -3.049 (n = 159, err = 650.0)
|   |   |   [13] draw_time < 9.5: -2.352 (n = 58, err = 239.4)

Number of inner nodes:    6
Number of terminal nodes: 7
```
